# The Pseudophosphatase MK-STYX Induces Neurite-Like Outgrowths in PC12 Cells

**DOI:** 10.1371/journal.pone.0114535

**Published:** 2014-12-05

**Authors:** Brittany M. Flowers, Lauren E. Rusnak, Kristen E. Wong, Dallas A. Banks, Michelle R. Munyikwa, Alexander G. McFarland, Shantá D. Hinton

**Affiliations:** Department of Biology, Integrated Science Center, College of William and Mary, Williamsburg, VA, United States of America; Temple University School of Medicine, United States of America

## Abstract

The rat pheochromocytoma PC12 cell line is a widely used system to study neuronal differentiation for which sustained activation of the extracellular signaling related kinase (ERK) pathway is required. Here, we investigate the function of MK-STYX [MAPK (mitogen-activated protein kinase) phosphoserine/threonine/tyrosine-binding protein] in neuronal differentiation. MK-STYX is a member of the MAPK phosphatase (MKP) family, which is generally responsible for dephosphorylating the ERKs. However, MK-STYX lacks catalytic activity due to the absence of the nucleophilic cysteine in the active site signature motif HC(X_5_)R that is essential for phosphatase activity. Despite being catalytically inactive, MK-STYX has been shown to play a role in important cellular pathways, including stress responses. Here we show that PC12 cells endogenously express MK-STYX. In addition, MK-STYX, but not its catalytically active mutant, induced neurite-like outgrowths in PC12 cells. Furthermore, MK-STYX dramatically increased the number of cells with neurite extensions in response to nerve growth factor (NGF), whereas the catalytically active mutant did not. MK-STYX continued to induce neurites in the presence of a MEK (MAP kinase kinase) inhibitor suggesting that MK-STYX does not act through the Ras-ERK/MAPK pathway but is involved in another pathway whose inactivation leads to neuronal differentiation. RhoA activity assays indicated that MK-STYX induced extensions through the Rho signaling pathway. MK-STYX decreased RhoA activation, whereas RhoA activation increased when MK-STYX was down-regulated. Furthermore, MK-STYX affected downstream players of RhoA such as the actin binding protein cofilin. The presence of MK-STYX decreased the phosphorylation of cofilin in non NGF stimulated cells, but increased its phosphorylation in NGF stimulated cells, whereas knocking down MK-STYX caused an opposite effect. Taken together our data suggest that MK-STYX may be a regulator of RhoA signaling, and implicate this pseudophosphatase as a regulator of neuronal differentiation.

## Introduction

PC12 cells serve as a model for neuronal cell differentiation because they differentiate in response to neurotrophins [1]–[4]. Moreover, they respond to nerve growth factor (NGF) and epidermal growth factor (EGF) differently. While both growth factors require Ras activation to stimulate the ERK/MAPK (extracellular regulated kinase/mitogen-activated protein kinase) pathway [5], [6], NGF induces a sympathetic neuron-like phenotype, while EGF promotes proliferation [7]. The differential response in PC12 cells is widely accepted to be due to differences in the duration of MAPK activation. NGF causes sustained activation of MAPK, implying sustained MAPK phosphorylation [2], [8]–[10], whereas EGF causes transient activation, implying rapid dephosphorylation [11]-. Numerous studies support a model in which sustained MAPK activation is required for neuronal differentiation [21], [25], [29]. Furthermore, sustained MAPK activation is well characterized as an inducer of neuronal differentiation of PC12 cells [10], [14]. However, the mechanisms of PC12 neuronal differentiation are incompletely understood. Other signaling pathways, such as the Rho/ROCK (RhoA kinase) pathway, have been implicated in PC12 differentiation as well [2], [15]–[17].

The duration and extent of MAPK activation depends on the activity of both kinases and phosphatases that regulate MAPK signaling [18]–[20]. Most MAPKs are substrates for members of the dual specificity protein tyrosine phosphatases that hydrolyze phospho-serine, -threonine, and -tyrosine residues on MAPK, and thus are termed MAPK phosphatases (MKPs) [21]. The protein tyrosine phosphatase SHP-2 is required for Ras and MAPK activation and neuronal outgrowth [22], [23]. Furthermore, the dephosphorylation of ERK1/2 MAPK by MKPs plays an important role in ERK signaling in both time and space [24]. Three MKPs, MKP-1, MKP-2, and MKP-3, have been implicated in neuronal differentiation and are expressed in a wide variety of tissues and cell types, including PC12 cells. Intriguingly, both EGF and NGF elicit a rapid increase in MKP-1 and MKP-2 mRNAs levels in PC12 cells [25], whereas only NGF induces a rapid increase in MKP-3 mRNA levels [26]. Although all three MKPs inhibit MAPK activation, MKP-3 has been studied more extensively in neuronal cells [25]–[27]. MKP-3 is significantly up-regulated and sustained for five days in PC12 cells stimulated by NGF [26], and is a known regulator of the duration of MAPK activation [28]. However, this does not address whether the rapid production of MKP leads to the transient MAPK response to EGF.

The prototypical catalytically inactive pseudophosphatase, STYX (serine threonine tyrosine interacting protein), has a glycine residue at the position expected for the active-site cysteine residue [29]. Initially it was proposed that STYX represented a new class of pSer/pThr/pTyr-binding proteins that function as dominant negative antagonists of endogenous protein phosphatases [30]. STYX associates with the spermatid phosphoprotein CRHSP-24 (calcium-responsive heat-stable protein with a molecular mass of 24k Da), which is a unique RNA binding protein. STYX knockout mice are defective for sperm production, revealing an essential function in spermatogenesis [31]. During neuronal differentiation, STYX competes with MKP-2 for binding to ERK1/2 MAPKs [32]. Moreover, it reduces activation of ERK1/2 MAPK and, thereby, blocks PC12 differentiation [32]. Considering that STYX is not a member of the MKP family, its role in the ERK signaling pathway was unexpected. In fact the interaction between STYX and ERK signaling was pursued only because of computational modeling based on the ERK/MAPK activation by MEK (MAP kinase kinase) [32]. Thus, we asked what would be the role of a pseudophosphatase that is a member of the MKP family in neuronal differentiation?

The pseudophosphatase MK-STYX is a product of the *DUSP24* gene, which has homology to the *MKP-3* gene [33]. However, MK-STYX is a unique member of the MKP family [34]. The active site signature motif **HC**(X_5_)**R** is essential for phosphatase activity, but MK-STYX has the sequence I**FS**TQGIS**R**S, which renders it catalytically inactive [30], [33], [34]. Mutations made in MK-STYX that restore this signature motif generate an active phosphatase (MK-STYX_active_) [34]. Despite being catalytically inactive, MK-STYX plays a role in a number of important cellular pathways [34]–[36], including inhibition of apoptosis and stress granule formation [34], [35], [37]. The functionality of MK-STYX is underexplored and still remains elusive; however, as with active MKP homologs, determining its interaction partners has helped elucidate some of the functions of MK-STYX [34], [38]. In earlier studies concerning the cellular function of MK-STYX, we discovered that it binds G3BP-1 (Ras-GTPase activating protein SH3 domain binding protein-1), which regulates both the Ras signaling pathway and stress granule assembly [1]. We determined that MK-STYX inhibits stress granule formation [34]. Dephosphorylation of G3BP-1 at Ser149 is critical for stress granule formation [39]; however, MK-STYX's interaction with G3BP-1 and its effect on the stress granule pathway is independent of the phosphorylation status of Ser 149 [37].

Given the apparent role of STYX in MAPK activation, this study focused on the role of MK-STYX in PC12 cell differentiation. Opposite to the effects of STYX, MK-STYX, but not the catalytically active mutant, induced neurite-like outgrowths. MK-STYX dramatically increased the number of cells with neurite extensions in response to NGF, whereas MK-STYX_active_ decreased the number of cells with extensions. MK-STYX continued to induce neurites in the presence of a MEK inhibitor. These data suggest that MK-STYX does not function through the ERK/MAPK signaling pathway like STYX does, confirming previous findings in our lab, and a report that MK-STYX has no effect on ERK activation [35]. Rather, we show that MK-STYX induced extensions through the Rho signaling pathway. MK-STYX overexpression decreased RhoA activation, and RhoA activation increased when MK-STYX was down-regulated, Moreover, MK-STYX effected downstream targets such as cofilin, which has a major role in actin filament networks [40]. MK-STYX decreased cofilin phosophorylation in non-stimulated cells, but increased cofilin phosphorylation 24 hours after NGF stimulation. Taken together, our data provide evidence that MK-STYX acts as a regulator in the Rho signaling pathway and neuronal differentiation.

## Materials and Methods

### Cell Culture and Transient Transfection

PC12 (rat pheochromocytoma) cells (ATCC) were maintained at 37°C, 5% CO_2_ in Roswell Memorial Institute (RPMI) medium (Gibco, Invitrogen) supplemented with 10% horse serum (Invitrogen) and 5% fetal bovine serum (FBS) (Invitrogen) or high glucose Dulbecco's Modified Eagle Medium (DMEM) (Gibco, Invitrogen) supplemented with 10% FBS. Using Lipofectamine 2000 (Invitrogen), cells were transfected with expression plasmids pMT2, pMT2-FLAG-MK-STYX-FLAG, or pMT2-FLAG-MK-STYX_active_-FLAG and pEGFP. Cells were either not stimulated or stimulated with nerve growth factor (NGF), or treated with a MEK inhibitor for the subsequent experiments, and analyzed with fluorescence microscopy.

### Live Cell Imaging and Scoring Cells

Cells were seeded at 1.5×10^5^ cells in a 60 mm dish (Fisher) and transfected 16 or 24 hr post seeding. Live cell imaging of EGFP-expressing cells was conducted with phase contrast and fluorescence microscopy using a Nikon ECLIPSE Ti inverted fluorescence microscope. Cells were observed over a five day period. Cells were scored either day 3 or 5 post-treatment for “neurites,” defined as neurite-like outgrowths ≧20 µm in length. Cells were scored at least by day 3, when the outgrowths were clearly visible. Neurite outgrowth length was measured with NIS-Elements Basic Research software (version 3.10, Nikon). At least three replicate transfections were performed and at least 100 cells were scored per replicate. Samples were scored blind with regard to treatment and were scored independently by at least two different individuals. Cells were scored into two categories: no neurites and neurites, except for the initial experiments where cells were categorized as <20, 20–40, 40–60, 60–80, 80–100, 100+ µm. Since this experiment showed neurite extensions longer than 20 µm to be characteristic of differentiation, subsequent studies focused on cells producing extensions >20 µm.

### NGF Stimulation

Twenty-four hr post-transfection, PC12 cells were serum-starved in RPMI supplemented with 1% horse serum or DMEM with no serum for 8–12 hr and then stimulated with 100 ng/ml of NGF (Prospec). For time-dependent NGF stimulation, cells were lysed at 0 min, 1 min, 3 min, 5 min, 12 min, 30 min, 24 hr, and 48 hr.

### Immunoblotting

PC12 cells were transfected with pMT2-FLAG-MK-STYX-FLAG, or MK-STYX specific shRNA expression plasmids, lysed, and analyzed by western blotting. Cells were harvested in lysis buffer (50 mM HEPES, pH 7.2, 150 mM NaCl, 10% glycerol, 10 mM NaF, 1% Nonidet P-40 alternative [Calbiochem], and protease inhibitor cocktail tablets [Roche]). Lysates were centrifuged at 14,000×g for 10 min, and the supernatant protein concentration was determined by NanoDrop quantification. Lysates were resolved by 10% SDS-PAGE or 12% SDS-PAGE (cofilin experiments) and transferred to PVDF membrane by iBlot (Invitrogen) for immunoblot analysis with anti-FLAG (Sigma), anti-STYXL1 (antibody against MK-STYX, Sigma), phospho-cofilin (Cell Signaling), cofilin (Cell Signaling) or anti-ß tubulin (Pierce) antibodies, followed by chemiluminescent detection. When warranted, blots were stripped (200 mM glycine, 3.5 mM SDS, 1% Tween 20), and re-probed.

### MK-STYX Knockdown

Control or MK-STYX-specific shRNA expression plasmids (Qiagen) were transfected into PC12 cells for 24 hr. RNA was isolated from the cells with RNAzol (MRC). cDNA synthesis was performed with RT^2^ First Strand Kit (Qiagen), and real time PCR was performed with RT^2^ SYBER Green ROX qPCR Mastermix (Qiagen) and RT^2^ qPCR Primer Assay [primers specific for STYXL1 (MK-STYX)] (Qiagen). Gene expression was tracked in real time using the Applied Biosystems StepOne Real-Time PCR-System. Triplicates were performed to determine the optimal amount of MK-STYX shRNA. Knockdown was also further analyzed by immunoblotting. Twenty-four hr post-transfection cells were stimulated with 100 ng/ml NGF and analyzed for neurite outgrowth.

### MEK Inhibition

The MEK inhibitor PD98059 (Cell Signaling) was reconstituted in DMSO according to the manufacturer's protocol. Cells were seeded at 1.5×10^5^ in a 60 mm dish and transfected 16 or 24 hr post seeding. 24 hr post-transfection cells were treated with 50 µM PD98059 for one hour and then stimulated with 100 ng/ml NGF for 3 days (media was replaced every 24 hr with treatment media), and live cell images were taken. Experiments were performed four times.

### Rho Activity Assays

Cells were stimulated at various time points with NGF, harvested with lysis buffer, immediately snap-frozen with liquid nitrogen, and stored at −80°C until Rho activity assays were performed. The Cytoskeleton Inc. manufacturer's protocol for RhoA G-LISA or RhoA ELISA was followed. RhoA G-LISA and ELISA were also performed for knockdown experiments. RhoA ELISA was used to normalize the RhoA G-LISA assay. Three replicate experiments were performed per assay.

## Results

### MK-STYX induces neurite outgrowth formation in PC12 cells

**Figure 1:**
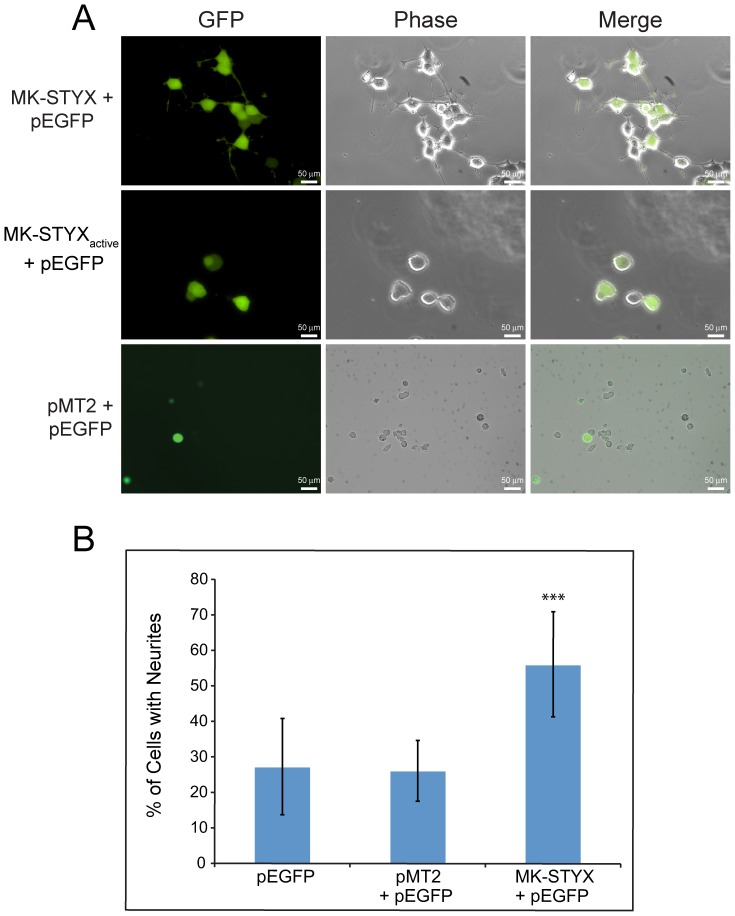
Pseudophosphatase MK-STYX induces neurite extensions in PC12 cells. (**A**) Representative examples are presented to illustrate neurite outgrowths of PC12 cells over-expressing MK-STYX and GFP, MK-STYX_active_, or pMT2 control plasmid and GFP. Cells were incubated 5 days. (**B**) Cells transfected with pEGFP, pMT2, or pMT2-FLAG-MK-STYX-FLAG plasmids were scored for neurite extensions ≥20 µm with a phase objective. Three replicate experiments were performed (n = 100 cells per experiment); the results are ± SEM. Statistical analysis was performed using ANOVA (F_2,8_ = 76.10, ***p<0.0001).

Since we previously identified G3BP-1 as an interacting binding partner of MK-STYX [34], and another pseudophosphatase, STYX, inhibits PC12 cell differentiation, we sought to determine whether MK-STYX has an effect on neuronal signaling by using PC12 cells as the model. To determine whether MK-STYX induces or inhibits neurite formation, PC12 cells were transfected with pEGFP and pMT2, MK-STYX, or MK-STYX_active_ and observed for neurite outgrowth. By day 5, the results were striking; cells transfected with MK-STYX and pEGFP developed neurites, whereas those transfected with MK-STYX_active_, pMT2 or pEGFP did not (Figure 1A). Significantly more of the cells transfected with MK-STYX and pEGFP (∼55%) had neurites, as compared to control cells expressing the empty pMT2 and pEGFP vectors (∼22%) or pEGFP alone (∼25%) (ANOVA: F_2,8_ = 76.10, p<0.0001) (Figure 1B). These outgrowths were a result of expressing MK-STYX alone.

### MK-STYX enhances the effects of NGF

**Figure 2:**
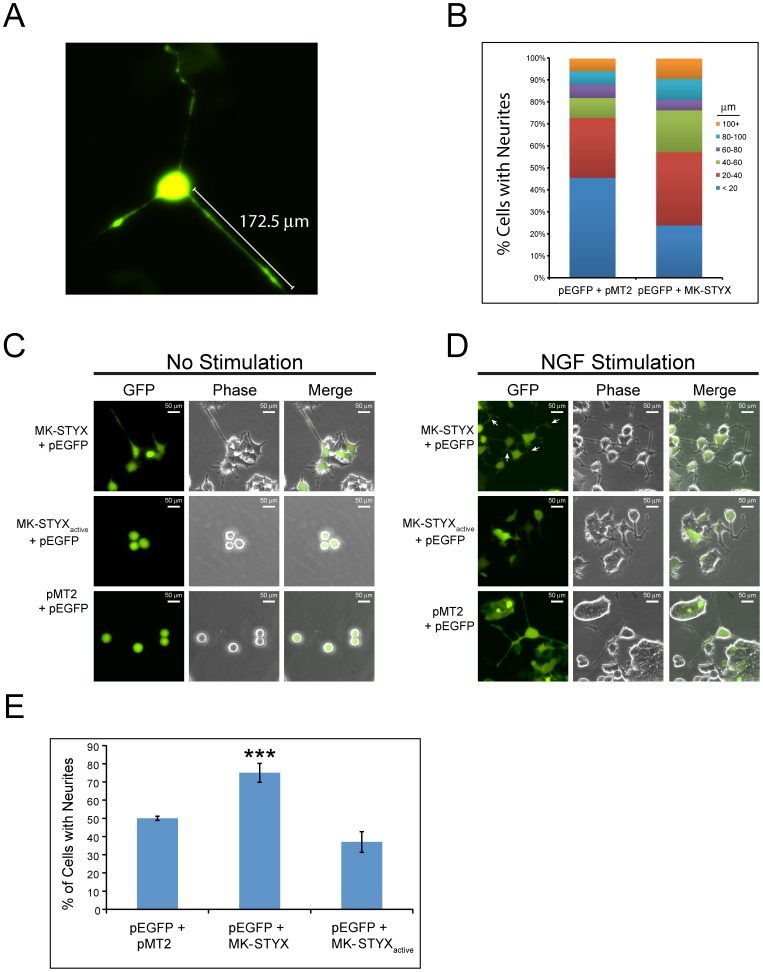
MK-STYX shifts the distribution of neurite outgrowth lengths. (**A**) The length of a neurite-like outgrowth of PC12 cells over-expressing GFP and MK-STYX was measured to demonstrate how cells were scored for outgrowths ≧20 µm. (**B**) PC12 cells over-expressing GFP and MK-STYX or pMT2 control plasmid for 24 hr were stimulated with 100 ng/ml NGF. Twenty-four hr post-stimulation cells were scored (n = 200) for outgrowth length. Cells were (**C**) not stimulated or (**D**) stimulated with 100 ng/ml of NGF. White arrows indicate “branching out” of primary neurites. (**E**) Cells were scored for neurite extensions ≧20 µm, and statistical analysis was performed using ANOVA (F_2,8_ = 43.81, ***p<0.001).

To determine the PC12 outgrowth lengths, cells were imaged and the extensions were measured from the body to the end as depicted in Figure 2A, where the neurite-like outgrowth measured was 172.5 µm. As the standard in subsequent experiments, an outgrowth ≧20 µm was considered to be a neurite-like outgrowth. To analyze MK-STYX's effects on normal cellular signaling in response to growth factors further, we stimulated cells co-expressing pEGFP and pMT2 or MK-STYX with NGF. NGF caused neurite extension in all cells, but MK-STYX shifted the distribution to much longer outgrowths (Figure 2B).

To examine the effect of MK-STYX on NGF-stimulated PC12 cells further, we also analyzed the active mutant, which is capable of dephosphorylation [34], [37]. Unlike MK-STYX, MK-STYX_active_ did not induce neurites (Figure 2C), but reduced them significantly (37%) (ANOVA (F_2,8_ = 43.81, p<0.001) in NGF stimulated cells (Figure 2D) as compared to wild-type MK-STYX (∼79%), or the control (50%) (Figure 2E). Neurites in cells transfected with MK-STYX and stimulated with NGF branched more (Figure 2D) than either the control or non-stimulated cells over-expressing MK-STYX. The neurite-like extensions of these cells separated further (depicted by white arrows). When primary neurites (extension connected to the cell body) divide further, this process is referred to as “branching out” [41].

### MK-STYX is required for NGF stimulated PC12 neurites

**Figure 3:**
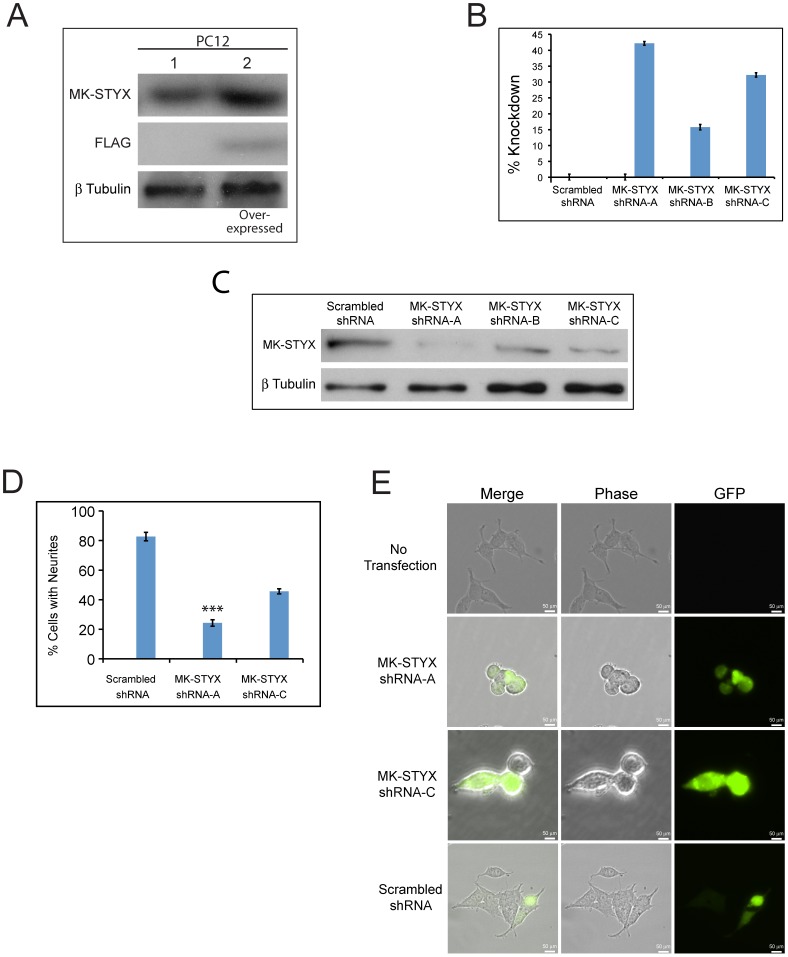
Knockdown of MK-STYX prevents NGF stimulated PC12 neurite extensions. (**A**) PC12 cells were transfected with pMT2-FLAG-MK-STYX-FLAG. Transfected and non-transfected PC12 cells were lysed and immunoblots performed. Blots were probed with anti-STYXL, to detect endogenous MK-STYX, and anti-FLAG to detect over-expressed MK-STYX. (**B**) PC12 cells were transfected with shRNA against MK-STYX. Quantitative RT-PCR analysis of MK-STYX mRNA levels after knockdown with three specific shRNAs targeting unique regions of MK-STYX. MK-STYX shRNA-A provided the best knockdown of endogenous MK-STYX at 42%, compared to Clone B (MK-STYX-shRNA-B) (∼15%) or Clone C (MK-STYX-shRNA-C) (∼32%). (**C**) PC12 cells transfected with control, MK-STYX shRNA-A, or MK-STYX shRNA-C were lysed and immunoblotted. Anti-STYXL1 antibody showed that endogenous MK-STYX was down-regulated by both MK-STYX shRNA-A and MK-STYX shRNA-C relative to the scrambled negative control. The blot was stripped and probed with anti-ß tubulin as a loading control. Replicate experiments were performed. (**D**) Cells expressing negative control, MK-STYX shRNA-A, or MK-STYX shRNA-C were scored for neurite extensions ≥20 µm. Three replicate experiments were performed (n = 100 cells per experiment); error bars indicate ± SEM. Statistical analysis was performed using ANOVA (F_2,8_ = 357.85, ***p<0.0001). (**E**) Representative examples of PC12 cells over-expressing shRNAs against MK-STYX (MK-STYX shRNA-A, MK-STYX shRNA-C, or scrambled negative control. The shRNA expression plasmids co-express GFP, which allows visualization of successful transfection. 24 hr post-transfection cells were stimulated with 100 ng/ml NGF, and 72 hr post-stimulation images were taken with phase contrast or fluorescence microscopy. Untransfected cells treated with NGF served as a positive control for neurite outgrowth.

First, to determine whether MK-STYX is endogenously expressed in PC12 cells, cell extracts were subject to immunoblot analysis. A single band on blots probed with anti-STYXL1 confirmed that MK-STYX is endogenously expressed (Figure 3A, lane 1). In PC12 cells over-expressing FLAG-tagged MK-STYX, the exogenous proteins were detected by both anti- STYXL1 and anti-FLAG (Figure 3A, lane 2).

Next, to determine the effects of MK-STYX specific expression plasmids, cells were transfected with various clones. Cells transfected with MK-STYX shRNA Clone A (MK-STYX shRNA-A) or Clone C (MK-STYX shRNA-C) showed the greatest knockdown of MK-STYX mRNA levels, at 42% and 32%, respectively (Figure 3B), as well as knockdown of protein levels (Figure 3C). To determine the importance of MK-STYX in neurite outgrowths in PC12 cells, cells were transfected with shRNAs against MK-STYX, stimulated with NGF, and observed for neurite outgrowths. Eighty two percent of cells expressing the scrambled shRNA negative control had neurite outgrowths. In contrast, only 24% of cells expressing MK-STYX shRNA-A and 46% of cells expressing MK-STYX shRNA-C formed neurites (Figure 3D), providing evidence that MK-STYX is important for neurite outgrowth in PC12 cells. Statistical analysis was performed using ANOVA (F_2,8_ = 357.85, p<0.0001). Furthermore, there was a dramatic difference in the phenotype in cells where MK-STYX had been down-regulated (Figure 3E). Cells expressing MK-STYX shRNA-A or MK-STYX shRNA-C appeared more rounded, whereas those expressing the scrambled control formed neurites similar in morphology to non-transfected cells (Figure 3E) that were used as a positive control.

### MK-STYX induces neurites in the presence of a MEK inhibitor

**Figure 4:**
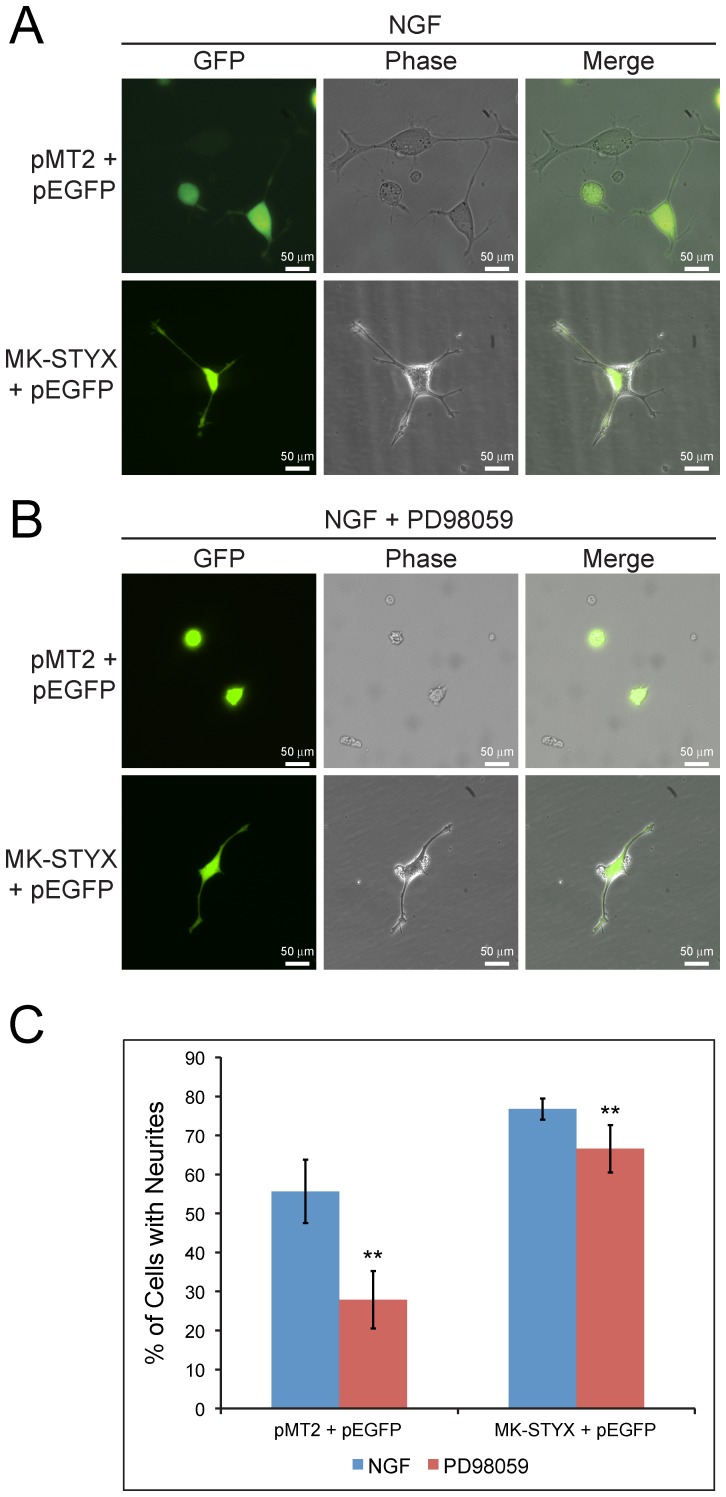
MK-STYX induces neurites in the presence of MEK inhibitor (PD98059). Representative examples of PC12 cells over-expressing pMT2 and GFP, or MK-STYX and GFP, that were stimulated with 100 ng/ml NGF and (**A**) treated with, or (**B**) not treated with 50 µM PD98059. (**C**) Cells were scored for neurite extensions ≧20 µm. Statistical analysis was performed with t-tests for control group (pMT2 and pEGFP; **p<0.01) and experimental group (MK-STYX and pEGFP; **p<0.01).

To determine whether MK-STYX induces neurites in PC12 cells through the ERK/MAPK pathway, MEK, which is necessary for ERK/MAPK activation [42], was blocked by PD98059, a MEK-specific inhibitor. Neurites formed in cells coexpressing pMT2 and pEGFP, or MK-STYX and pEGFP (Figure 4A and C), but in the presence of the inhibitor the formation of neurites decreased by 50% (Figure 4B and C) in the control cells expressing pMT2 and pEGFP stimulated with NGF (t-test; p<0.01). In contrast, MK-STYX maintained its ability to induce neurites in the presence of PD8059 (Figure 4B and C). The fact that MK-STYX induced neurites in the presence of MEK (t-test; p<0.01), suggests that MK-STYX acts further downstream in the ERK/MAPK pathway or through a different pathway to induce neuronal differentiation in PC12 cells. Furthermore, MK-STYX did not affect Ras activity (data not shown), and immunoblot analysis demonstrated that ERK activation was not affected by MK-STYX (data not shown), consistent with our previous data from Cos-1 cells (unpublished), and reported findings in HeLa cells [35].

### MK-STYX decreases RhoA activation

**Figure 5:**
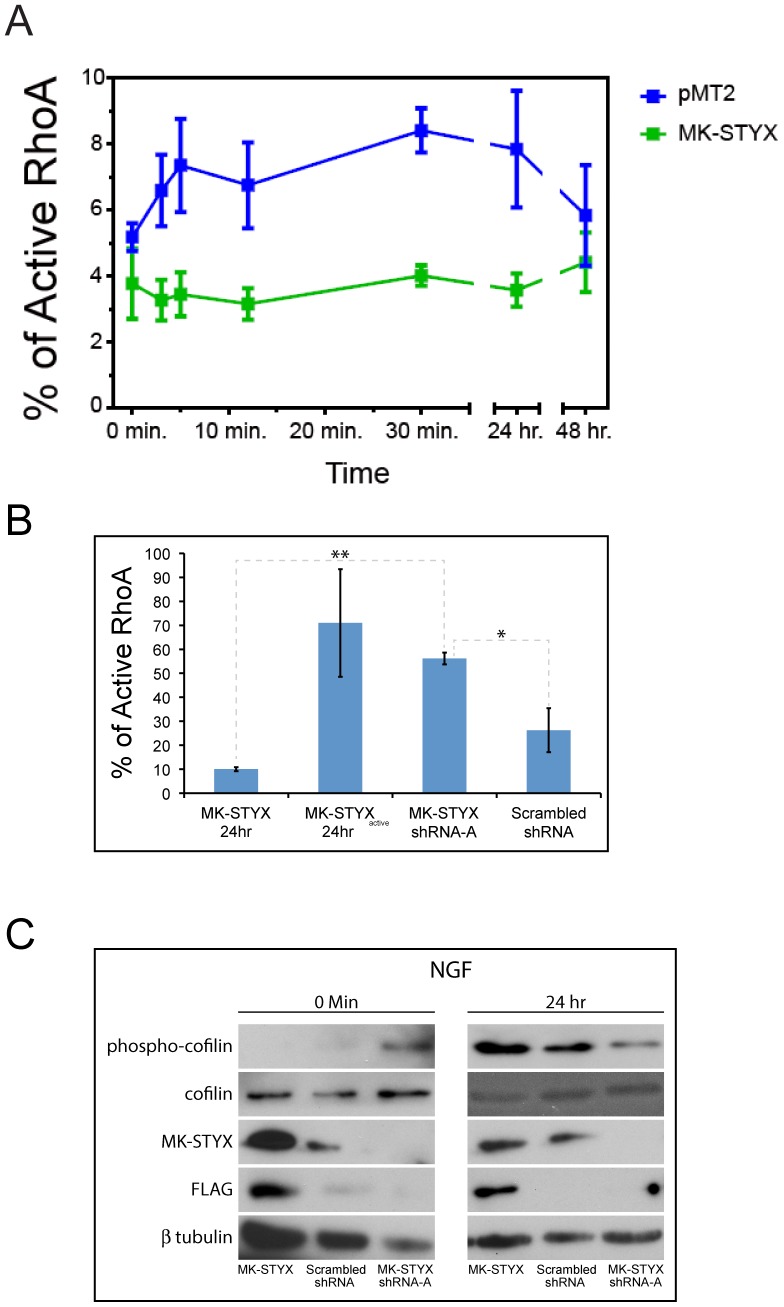
MK-STYX decreases RhoA activation. (**A**) PC12 cells were transfected with pMT2 or pMT2-FLAG-MK-STYX-FLAG. 24 hr post-transfection cells were stimulated with 100 ng/ml NGF, and lysed at the indicated time points. Activation of RhoA was quantified by RhoA G-LISA small G-protein assay. Total RhoA was normalized by RhoA ELISA. Three replicate experiments were performed. Error bars indicate ± SEM. Statistical analysis was performed using multiple t-tests, and there was a significant increase in the percentage of active RhoA in pMT2-expressing control cells within 30 minutes (p<0.05) (**B**) PC12 cells were transfected with pMT2-FLAG-MK-STYX-FLAG, MK-STYX shRNA-A, or scrambled shRNA. 24 hr post-transfection cell were stimulated with 100 ng/ml NGF, lysed, and RhoA activation was quantified by RhoA G-LISA small G-protein assay. Total RhoA was normalized by RhoA ELISA. Three replicate experiments were performed. Error bars indicate ± SEM. Statistical analysis was performed using ANOVA (F_3.9_ = 5.066, **p<0.01; *p<0.05). (**C**) PC12 cells transfected with MK-STYX, scrambled shRNA, or MK-STYX shRNA-A. 24 hr post-transfection cells were stimulated with NGF or not, and were lysed 24 hr thereafter and immunoblotted. Anti-phospho-cofilin antibody showed that MK-STYX decreased cofilin phosphorylation in non-stimulated cells relative to the MK-STYX shRNA-A. However, MK-STYX increased cofilin phosphorylation in cells stimulated with NGF relative to the MK-STYX shRNA-A. These blots were stripped and probed for cofilin as a loading control. Anti-STYXL1 antibody showed over-expressed MK-STYX relative to the scrambled control, and that endogenous MK-STYX was down-regulated by MK-STYX shRNA-A relative to the scrambled negative control. The blot was stripped and probed with anti-FLAG to detect over-expressed MK-STYX, and probed for anti-ß tubulin as a loading control. Three replicate experiments were performed.

We performed Rho activation assays to test further whether MK-STYX functions independently of the ERK/MAPK pathway. Inactivation of Rho has been shown to be required for neurite induction in PC12 cells [16]. Strikingly, MK-STYX prevented RhoA activation (Figure 5A), and levels of active RhoA remained low and consistent throughout the duration of the experiment. In contrast, there was a significant increase in the percentage of active RhoA in control cells within 30 minutes (p<0.05); activation was sustained for 24 hr and then declined back to baseline levels after 48 hr.

RhoA has multiple roles in neuronal differentiation of PC12 cells, and its inactivation is required during the initial stages of differentiation [16]. This commitment of PC12 cells usually occurs within 24–48 hr, after RhoA activity has declined to minimal levels [43], [44].

We performed knockdown studies to determine the effect of down-regulation of MK-STYX on Rho activation. Because cells expressing MK-STYX shRNA-A showed the greatest decrease in MK-STYX levels (see Figure 3B and C), this shRNA construct was used in the RhoA activation experiment. To understand the dynamics of MK-STYX further, the active mutant was also included in these studies. Cells over-expressing MK-STYX_active_ stimulated with NGF for 24 hr had a dramatic increase in RhoA activation compared to cells over-expressing MK-STYX stimulated with NGF for 24 hr or scrambled control (Figure 5B). However, when endogenous MK-STYX was knocked down by MK-STYX shRNA-A, RhoA activation dramatically increased compared to cells over-expressing MK-STYX stimulated with NGF for 24 hr or the scrambled control (Figure 5B) [ANOVA (F_3,9_ = 5.066, p<0.01)]. These findings suggest a role for MK-STYX in regulating Rho activity.

### MK-STYX affects RhoA downstream target cofilin

Since we showed that MK-STYX inactivates RhoA, the question arose whether MK-STYX has an effect on downstream targets of RhoA. To address this question we analyzed phosphorylation of cofilin, an actin binding protein that is regulated by RhoA. Cells were transfected with MK-STYX, scrambled control, or MK-STYX shRNA-A and either stimulated with NGF for 24 hr or not. Cofilin phosphorylation decreased in cells over-expressing MK-STYX or scrambled control in unstimulated cells, compared with the levels of phospho-cofilin present shRNA-A-expressing cells. However, 24 hr post NGF stimulation, cofilin phosphorylation increased in cells over-expressing MK-STYX. Taken together, these data suggest that MK-STYX may play an important role in cofilin activation and inactivation, and in the actin filament network.

## Discussion

There is an abundant population of human pseudoenzymes within the kinase and phosphatase families that may play key roles in signaling cascades [45], [46]. Yet, nearly two decades after the discovery of the prototypical pseudophosphatase, STYX [29], [30], little is known about the functions or molecular mechanisms of the majority of the pseudophosphatases. The *C. elegans* EGG pseudophosphatases serve as the best mechanistic examples; EGG4 and EGG5 bind and regulate signaling through the dual-specificity tyrosine-phosphorylated and -regulated kinase family mini brain kinase 2 [45], [47]. Previous studies have shown that MK-STYX is a master regulator of apoptotic potential of the mitochondria [35]. Knockdown of MK-STYX blocks release of cytochrome c, and prevents cells from undergoing apoptosis [35]. Recently, a model was provided for the molecular mechanism by which MK-STYX controls apoptosis, suggesting that MK-STYX negatively regulates PTPM1 (PTP localized to the mitochondrion 1). MK-STYX interacts with PTPM1 and inhibits its catalytic activity, regulating cell viability [38]. Such interactions between a pseudophosphatase and an active phosphatase are also seen among the myotubularin phosphatases [38]. However, binding of the myotubularin pseudophosphatase to its active homolog enhances phosphatase activity [48]. Previously, we reported that MK-STYX has a role in the stress response pathway by inhibiting stress granule formation [34], [37]. In this study, we report that it is important for neurite outgrowth in PC12 cells and decreases RhoA activation.

It has been suggested that pseudophosphatases serve a “dominant-negative” function by physically masking the phosphorylated residues, thereby blocking access of phosphatases, and protecting substrates from dephosphorylation [45], [49]. Thus, it seemed plausible that a pseudophosphatase might have a role in sustaining the MAPK activation required for PC12 neuronal differentiation. Findings with STYX have shown that this pseudophosphatase competes with MKP-2 to bind ERK1/2 MAPK [32]; however, STYX's interaction with ERK1/2 MAPK is much more dynamic than merely serving as a blocker. STYX acts as a nuclear anchor for ERK1/2 MAPK, thereby regulating its activation [32]. Furthermore, over-expression of STYX prevents PC12 differentiation [32].

In contrast, we show that over-expressing MK-STYX induces neurite-like outgrowths, a hallmark of PC12 differentiation. In addition to inducing outgrowths, when cells expressing MK-STYX were stimulated with NGF, longer neurites formed and more branching occurred, suggesting these cells are becoming neuronal. By analogy with the STYX results, we thought that MK-STYX might exert its effects on PC12 differentiation through the Ras-ERK/MAPK pathway. However, our studies with the MEK inhibitor provide evidence that MK-STYX does not function through Ras-ERK/MAPK, but further downstream or by another pathway. Furthermore, we have observed that MK-STYX does not have an effect on Ras activation in the presence or absence of NGF (unpublished observations). Other studies have reported that NGF-dependent survival of PC12 cells may be mediated by other pathways such as the phosphatidylinositol-3-kinase pathway [50], [51]. Here, we show that MK-STYX is involved in the RhoA signaling pathway, which also has been implicated in PC12 neuronal differentiation [16]. Further support for a role of MK-STYX in the RhoA signaling pathway comes from the branching out pattern seen in the presence of MK-STYX and NGF. This branching out pattern may represent the formation of dendrites [52], which are important for neuronal communication [41], [53], [54]. Furthermore, these extensions appear to overlap, suggesting that MK-STYX may have a role in branching [41], which is regulated by the Rho GTPases.

In addition, MK-STYX affected the RhoA downstream target cofilin, Cofilin has a dual role in the actin filament network, to depolymerize and/or sever actin [55], [56]. When phosphorylated, cofilin is inactive, resulting in filament stability, whereas dephosphorylation of cofilin leads to depolymerization [56]–[58] Thus, cofilin is a modulator of the actin cytoskeleton architecture, and plays a pivotal role in the rearrangement of the cytoskeleton that is required for neuronal differentiation. We demonstrate that MK-STYX decreased cofilin phosphorylation in the absence of NGF, which is required for the induction of neurites [59]. However, MK-STYX increased cofilin phosphorylation 24 hr post- NGF stimulation, similar to the commitment period of PC12 cells to become neuronal and begin branching [43], [44]. This supports the effects of MK-STYX on RhoA activation beyond the 24 hr NGF stimulation, providing more substantial evidence that MK-STYX has a role in RhoA signaling.

The Rho family of small GTPases has been implicated in reorganization of the actin cytoskeleton and subsequent morphological changes in various cell types [60], [61]. Rho family members serve as molecular switches by cycling between an inactive GDP-bound state and an active GTP-bound state. At least two members within this family, RhoA and RhoE, have been reported to have important roles in PC12 neurite extension [62], [63]. RhoE causes neurite outgrowths in PC12 cells by inhibiting the RhoA/ROCK-1 (Rho kinase) signaling cascade [62], and RhoA inactivation is essential for PC12 neurite outgrowth [63]. Our results have revealed a new component in the RhoA signaling pathway. We demonstrate here that MK-STYX decreases RhoA activation, while down-regulation of MK-STYX increases RhoA activation. Considering that G3BP-1, the first identified binding partner of MK-STYX [34], has been implicated to have a role in Rho signaling pathways [64], [65], MK-STYX may be an important regulator in this pathway. Furthermore, G3BP-1 also interacts with other macromolecules such as tau mRNA and has been implicated in differentiation of neuronal cells [52], [66]. Important future studies include determining whether the neurite-like outgrowths induced by MK-STYX differentiate into neurons, further analyzing MK-STYX's effects on branching, its molecular mechanism in PC12 differentiation, and the mechanism by which MK-STYX inhibits Rho activation. For example, MK-STYX may interact with the low molecular weight protein tyrosine phosphatase (LMW-PTP), p190GAP, or RhoA to inhibit its activity and induce neurite formation. There are several unanswered questions. However, pseudophosphatases have been shown to regulate phosphatase activity positively or negatively [38], [48]. Furthermore, MK-STYX could directly interact with RhoA, preventing its association with the membrane and rendering it inactive.

Various kinases and phosphatases have been implicated in controlling neuronal development and regeneration [67]. Moreover, it is becoming apparent that pseudokinases and pseudophosphatases are integral elements of signal transduction, and thus may exert important roles in the pathology of various diseases [68]. Although STYX was the first pseudophosphatase implicated in having a role in PC12 cell differentiation by regulating ERK1/2 activation, the present study provides the first evidence that MK-STYX has an effect very different from that of STYX in this process. Over-expression of MK-STYX resulted in PC12 cell differentiation, and knocking down MK-STYX prevented neurite formation, suggesting that the pseudophosphatase plays an important role in PC12 cell differentiation. However, MK-STYK differs from STYX's role as an ERK1/2 regulator, in that MK-STYX bypasses Ras-ERK/MAPK signaling or functions through another pathway. The precise mechanism underlying the effects of MK-STYX remains to be defined; however, results from this study support a mode of action of MK-STYX through the RhoA signaling pathway. Further characterization of the role of MK-STYX in neuronal cells may generate new insights into neuronal development and potential approaches to therapy for neurodegenerative diseases such as amyotrophic lateral sclerosis or Alzheimer's.
